# Diet quality of Japanese adults with respect to age, sex, and income level in the National Health and Nutrition Survey, Japan

**DOI:** 10.1017/S1368980019002088

**Published:** 2020-04

**Authors:** Kayo Kurotani, Kazuko Ishikawa-Takata, Hidemi Takimoto

**Affiliations:** Department of Nutritional Epidemiology and Shokuiku, National Institute of Health and Nutrition, National Institutes of Biomedical Innovation, Health and Nutrition, 1-23-1 Toyama, Shinjuku-ku, Tokyo 162-8636, Japan

**Keywords:** Diet quality, Socioeconomic status, Household income, Dietary guidelines, Japanese, National Health and Nutrition Survey, Japan

## Abstract

**Objective:**

Although several studies in Western countries show that higher socioeconomic status is associated with higher diet quality, no study has observed this association in Japan. In the current study, we examined the association between diet quality and the combinations of age, sex, and household income, and also compared the dietary intake between diet quality levels according to household income.

**Design:**

Cross-sectional study.

**Setting:**

National Health and Nutrition Survey, Japan in 2014.

**Participants:**

2785 men and 3215 women.

**Results:**

Higher Japanese Food Guide Spinning Top scores (better diet quality) were observed in older women, especially those with higher household income, whereas lower scores were observed in younger men with lower household income. Those having low quality diet, especially in low income households, had higher odds of not meeting the recommended amounts of the Japanese dietary guidelines, than those having high quality diet.

**Conclusions:**

Diet quality in Japanese adults differed by age and sex as well as by household income level. A different approach to diet quality improvement is needed according to population characteristics including not only age and sex but also social economic status.

Diet quality is affected not only by age and sex but also by socioeconomic status (SES) including income, occupation, and educational levels^(^^1^^)^. A review of cross-sectional studies in Western countries reported that high-SES individuals consume high quantities of whole grain bread, fruits, vegetables, lean meat, fish, milk, and dairy products; however, low-SES individuals consume high quantities of grains and starchy vegetables, meat, processed meat, eggs, fats, and sweets^(^^1^^)^. In the National Health and Nutrition Survey (NHNS) in Japan, low and middle income level households, compared with high income households, report a higher intake of cereals, and a lower intake of potatoes and starches, pulses, vegetables, fruits, mushrooms, fish and shellfish, milk, and seasonings and spices^(^^2^^)^. These results suggest differences in the food intake patterns between lower income household members in Japan and those in Western countries. Previous studies focused on the intake of individual foods and nutrients. However, it is difficult to identify the separate specific effects of individual foods and nutrients, because of confounding and interaction^(^^3^^)^. In contrast, diet quality indexes, which are summary measures of several foods or food groups, are used to overcome this difficulty.

Several studies in Western countries show that higher-SES is associated with higher scores of diet quality indexes^(^^4^^–^^6^^)^. However, no study examined the association between SES and diet quality in Japan. An updated meta-analyses showed that higher diet quality scores on the Healthy Eating Index (HEI), the Alternative Healthy Eating Index (AHEI), and the Dietary Approaches to Stop Hypertension were associated with lower risks of all-cause mortality, cardiovascular diseases, cancer, type 2 diabetes, and neurodegenerative disease^(^^7^^)^. In Japan, prospective studies reported that closer adherence to Japanese dietary guidelines was associated with lower risks of total mortality and mortality from cardiovascular diseases^(^^8^^,^^9^^)^. This evidence indicates that better diet quality was related to better health outcomes.

Several studies have shown that low-SES is associated with higher risks of mortality^(^^10^^,^^11^^)^ and severe diseases^(^^12^^,^^13^^)^. The improvement in diet quality among low-SES individuals may be effective in reducing health disparities. Here, we hypothesized that diet quality index was influenced by age, sex, and income level. In the current study, we examined the association between diet quality and the combinations of age, sex, and household income levels and also compared the dietary intake between diet quality levels, according to household income using the NHNS data.

## Methods

### Study procedure

The current cross-sectional study was based on data from the 2014 NHNS^(^^14^^)^ conducted by the Ministry of Health, Labour and Welfare. The NHNS has been running since 1945, and it is an annual nationwide survey based on the Health Promotion Law (Law No. 103, enacted in 2002), to assess the health status, food and nutrient intakes, and lifestyles of people living in Japan^(^^15^^)^. For the following age categories, the 2014 NHNS consists of: (i) physical examination (≥1 year); (ii) blood test (≥20 years); (iii) dietary survey (≥1 year); (iv) pedometer measurement (≥20 years); and (v) lifestyle questionnaire (≥20 years). The two-staged cluster randomized sampling method was applied for selecting the 300 sampling units (regions) for the NHNS, to cover all the 47 prefectures (the Japanese equivalent of provinces). These 300 units were randomly selected from the approximately 1000 census enumeration areas which participated in the preceding Comprehensive Survey of Living Conditions. Each unit is equivalent to two census enumeration areas. Subjects were household members aged ≥1 year (as at 1 November 2014), living in the 300 selected units. Of the 5432 eligible households in the units, dietary data were obtained from a total of 3648 households (response rate = 67·2 %). Based on official application procedures under Article 33 of the Statistics Act, unlinked anonymized NHNS data were only obtained with permission from the Ministry of Health, Labour and Welfare, Japan. Our study was conducted in accordance with the Ethical Guidelines of Epidemiological Research^(^^16^^)^.

### Dietary assessment

Dietary intake data were collected using a 1 d semi-weighted household dietary record, on an optional day in November, excluding holidays. A detailed description of the procedure has been published elsewhere^(^^14^^,^^15^^,^^17^^)^. All the foods and beverages consumed, food waste, leftovers, and foods consumed away from home were weighed and recorded in the dietary record, for each household. When food weight was missing, an official food item booklet with standard portion size for frequently consumed dishes was applied for estimation, which was conducted by trained dieticians. For shared dishes within the household, the approximate proportions of each food were assigned to individual household members for the estimation of the individual food intakes. Before the survey, trained dietitians from the public health centre demonstrated the measures to determine food quantities, and taught the survey methods and procedures to each person who usually cooks for the family. This member in each household was asked to record the names of food ingredients, weight, and the leftover amount of food for each individual household member. During the survey period, the dietitians visit each household at least once a day, to check the dietary record. Then, the average daily energy and nutrient intake per capita were calculated using the Japanese Standard Food Composition Table^(^^18^^,^^19^^)^.

### The Japanese Food Guide Spinning Top score

In the Japanese Food Guide Spinning Top, the amount of a certain dish that counts as one serving is defined for each dish category as follows^(^^20^^)^: one serving of a grain dish is composed of approximately 40 g of carbohydrates, one serving of a vegetable dish weighs about 70 g (uncooked), one serving of fish and meat dish amounts to about 6 g of protein, one serving of milk amounts to about 100 mg of calcium, and one serving of fruits weighs about 100 g. In addition, 100 % vegetable juice and 100 % fruit juice are counted as half the weight of the amount actually consumed. The recommended amount of servings for each dish category and the recommended total energy intake are specified according to sex, age, and physical activity level; the amount of energy intake from snacks and alcoholic beverages is recommended to be <200 kcal/d for all subgroups.

We determined the scores by measuring the adherence to the Japanese Food Guide Spinning Top from information in the dietary records (Supplementary Table 1). The procedure for creating an adherence score for the Japanese Food Guide Spinning Top has been described elsewhere^(^^8^^,^^9^^)^. We classified subjects who had exercise habits, spending ≥30 min, at least twice a week, as moderately physically active, and the remainder as sedentary. For vegetable dishes and fruits, we modified the original criteria (recommended range) to remove the upper limit of intake^(^^9^^)^. If individuals consumed the recommended amount of servings from any of the five dish categories or the recommended total energy, or energy from snacks and alcoholic beverages, 10 points were recorded for that group. If individuals exceeded or fell short of the recommended servings or energy, the score was calculated proportionately between 0 and 10 points. If an individual consumed less than the recommended amount of servings or energy, the score was calculated using the following formula: 10 × (the consumed amount of servings or energy)/(the lower limit of the recommended amount). If an individual consumed more than the recommended amount of servings or energy, the score was calculated using the following formula: 10 − 10 × [(the consumed amount of servings or energy) − (the upper limit of the recommended amount)]/(the upper limit of the recommended amount). Each score was rounded off to the nearest whole number. When this calculation produced a negative score due to excess servings or energy, the score was converted to 0. All group scores were summed to obtain a total Japanese Food Guide Spinning Top score ranging from 0 (the lowest adherence) to 70 (the highest adherence).

### Household income

Household income per year was elicited in the lifestyle questionnaire, with four options provided from which respondents could select (<2 million yen, 2–6 million yen, ≥6 million yen, and ‘Don’t know’)^(^^14^^)^. Instead of calculating household income per capita, we adjusted for household size in the statistical analyses, because we could not know whether each household member shared the answer about the household income. We defined the household income of <2 million yen as the low level of household income, 2–6 million yen as middle level, and ≥6 million yen as high.

### Study population

**Fig. 1:**
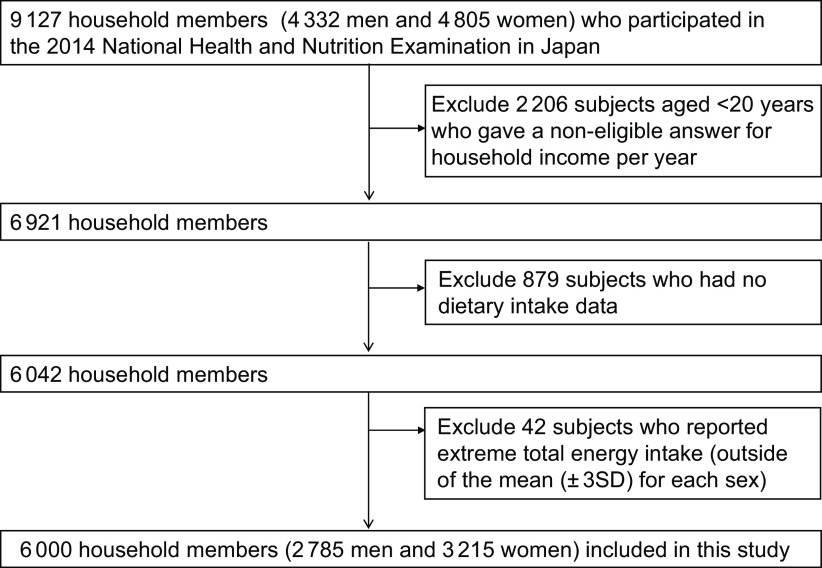
Participant flow chart

A participant flow chart is shown in Fig. 1. Of 9127 household members (4332 men and 4805 women), we excluded 2206 subjects aged <20 years, or those who chose ‘Don’t know’ or failed to give a correct answer (no answer or multiple answers) for household income per year, or when more than one person answered within the same household. Of 6921, we also excluded 879 subjects who had no dietary intake data. Furthermore, 42 household members who reported extreme total energy intake (outside of the mean (±3 sd) for each sex) were excluded. Ultimately, a total of 6000 household members (2785 men and 3215 women) were included in this study.

### Statistical analyses

Data were expressed as means (sd) and percentages for continuous and categorical variables, respectively, according to household income level. We calculated the multivariate adjusted scores of adherence to the Japanese Food Guide Spinning Top according to sex, age category (20–39 years, 40–59 years, and ≥60 years), and household income level (low, middle, and high). In the linear regression analysis, the trend associations were assessed by assigning the ordinal numbers 0–2 to the three categories of each household income level. We considered the following confounding variables: residential block (Hokkaido and Tohoku, Kanto, Hokuriku and Tokai, Kinki, Chugoku and Shikoku, and Kyushu and Okinawa), population size of residential area (Metropolitan area, city with population ≥150 000, and city with population <150 000), household size (1 person, 2 people, 3 or 4 people, and ≥5 people), one or more children aged under 15 years (yes or no), occupation (professional/manager, sales/service/clerical, security/transportation/labour, non-worker, and missing), BMI (<18·5, ≥18·5 and <25·0, and ≥25·0 kg/m^2^), smoking status (non-smoker, past smoker, current smoker, and missing), and habitual physical activity (yes or no).

Household members were divided into median of crude scores of adherence to the Japanese Food Guide Spinning Top by sex, age, and household income level subgroups. Individuals with median or higher scores were defined as the high quality diet group and the remainder as the low quality diet group. We calculated the multivariate adjusted means (95 % CI) of scores and intakes of each dish as well as those of intakes of nutrients that were related to lifestyle-related diseases^(^^21^^)^, as well as food groups according to diet quality level. We assessed the differences between high and low quality diet groups according to sex and household income level using an analysis of covariance. We did not show the differences between high and low quality diet groups according to age because we found no large differences by age subgroups. Additionally, we calculated the proportions of meeting the recommended amounts of each dish of the Japanese Food Guide Spinning Top according to the diet quality. We also calculated the proportions of meeting the tentative dietary goal for preventing lifestyle-related diseases (DG) in the dietary reference intake for Japanese (in 2015)^(^^21^^)^. Furthermore, we calculated the multivariate adjusted OR and 95 % CI of not meeting the Japanese Food Guide Spinning Top or DG. Because we focused on the low household income group, we further divided individuals into either the low-income households or the middle- and high-income households, in these analyses. Two-sided *P* value <0·05 was considered statistically significant in all analyses. All analyses were performed using SAS version 9.4 for Windows (SAS Institute).

## Results

Characteristics according to household income levels are shown in Table 1. The proportions of low, middle, and high levels of household income were 17 %, 56 %, and 27 %, respectively. Table 2 shows that higher scores of adherence to the Japanese Food Guide Spinning Top were observed in older women, especially those with higher household income, whereas the lower scores were observed in younger men with lower household income. In those aged 20–39, 40–59, and ≥60 years, respectively, the multivariate adjusted mean (95 % CI) scores were 45·5 (44·7, 46·4), 46·4 (45·7, 47·1), and 48·5 (48·0, 49·0) in men and 47·6 (46·9, 48·4), 48·9 (48·3, 49·5), and 51·4 (50·9, 51·9) in women (data not shown in table).

**Table 1:** Characteristics of household members according to household income level

	Household income (yen/year)			*P* for difference
	Low (<2 million)	Middle (2–<6 million)	High (≥6 million)	
*n*	1038	3370	1592	
Age (%)				
20–29 years	3·7	6·2	10·6	<0·0001
30–39 years	5·6	13·4	13·0	
40–49 years	7·4	14·0	21·7	
50–59 years	9·5	12·6	23·9	
60–69 years	27·0	26·9	15·3	
≥70 years	46·8	26·9	15·5	
Sex (%)				
Men	40·7	47·7	47·4	0·0002
Residential block (%)				
Hokkaido and Tohoku	19·9	14·4	11·3	<0·0001
Kanto	23·5	29·9	33·9	
Hokuriku and Tokai	10·7	15·8	19·5	
Kinki	18·7	16·1	17·6	
Chugoku and Shikoku	12·4	10·6	8·3	
Kyushu and Okinawa	14·8	13·2	9·4	
Population size of residential area (%)				
Metropolitan area	17·8	21·2	25·2	<0·0001
City with population ≥150 000	24·1	27·2	28·0	
City with population <150 000	58·1	51·7	46·8	
Occupation (%)*				
Professional/manager	5·1	10·5	28·6	<0·0001
Sales/service/clerical	17·2	25·1	26·6	
Security/transportation/labour	12·9	20·1	15·6	
Non-worker	64·7	44·4	29·2	
Household size (%)				
1 person	35·2	7·5	2·2	<0·0001
2 people	42·5	35·7	20·2	
3 or 4 people	18·4	43·3	57·2	
≥5 people	4·0	13·6	20·4	
Number of children aged under 15 years (%)				
0	93·4	78·6	70·2	<0·0001
1	3·4	8·9	15·5	
2	2·9	9·4	9·9	
3 or more	0·4	3·0	4·5	
Weight status (%)				
Underweight	22·7	24·1	25·2	0·02
Normal	53·5	56·2	56·2	
Overweight	23·8	19·6	18·6	
Smoking status (%)†				
Non-smoker	75·9	71·7	72·3	0·07
Past smoker	6·7	9·0	9·0	
Current smoker	17·4	19·3	18·8	
Physical activity (%)				
Habitual (≥2 d/week)‡	20·6	18·2	12·4	<0·0001

* Number of subjects was 5994.

† Number of subjects was 5953.

‡ Habitual physical activity was defined as spending 30 min or more engaged in moderate activity in two or more days per week for 1 year or more.

**Table 2:** Multivariate adjusted scores on adherence to the Japanese Food Guide Spinning Top according to sex, age, and household income level*

		Low household income (<2 million yen/year)		Middle household income (2–<6 million yen/year)		High household income (≥6 million yen/year)		*P* for trend
		Multivariate adjusted mean	95 % CI	Multivariate adjusted mean	95 % CI	Multivariate adjusted mean	95 % CI	
Men								
20–39 years	*n*	48		300		176		
		44·8	42·2, 47·4	44·6	43·6, 45·7	47·6	46·2, 49·0	0·02
40–59 years	*n*	69		405		349		
		45·6	43·5, 47·8	46·5	45·5, 47·4	46·7	45·6, 47·7	0·68
≥60 years	*n*	305		904		229		
		47·0	45·9, 48·0	48·8	48·2, 49·4	49·3	48·1, 50·4	0·004
Women								
20–39 years	*n*	48		359		200		
		48·7	46·2, 51·3	47·3	46·4, 48·3	48·1	46·8, 49·4	0·91
40–59 years	*n*	107		492		378		
		47·4	45·7, 49·1	48·4	47·6, 49·2	50·1	49·2, 51·1	0·003
≥60 years	*n*	461		910		260		
		50·6	49·7, 51·6	51·7	51·1, 52·3	51·2	50·0, 52·3	0·02

* Adjusted for residential block, population size of residential area, household size, one or more children aged under 15 years, occupation, BMI, smoking status, and physical activity.

Regardless of sex and household income level, individuals with a low quality diet (scores with less than the median) consumed lower amounts of vegetable dishes, milk, and fruits and had higher energy intake from snacks and alcoholic beverages, compared with those with a high quality diet (Table 3). Among men in the higher-income subgroups, those with a low quality diet consumed lower amounts of grain dishes. This positive association between diet quality and intake of grain dishes was also observed in women, regardless of household income level. Additionally, individuals with low quality diet had less energy intake from total carbohydrate and consumed less dietary fibre and potassium, compared with those with high quality diet; regardless of sex and household income level. Intake of sodium was inversely associated with diet quality among women in the low-income subgroup.

**Table 3:** Multivariate adjusted means and 95 % CI of the scores on the adherence to the Japanese Food Guide Spinning Top and the intakes of each dish category according to quality of diet*

	Low household income (<2 million yen/year)					Middle and high household income (≥2 million yen/year)				
	Low diet quality†		High diet quality†		*P* value	Low diet quality†		High diet quality†		*P* value
	Multivariate adjusted mean	95 % CI	Multivariate adjusted mean	95 % CI		Multivariate adjusted mean	95 % CI	Multivariate adjusted mean	95 % CI	
Men										
No. subjects	207		215			1174		1189		
Japanese Food Guide Spinning Top score	38·1	37·3, 38·9	54·0	53·3, 54·8	<0·0001	39·2	38·9, 39·5	54·1	53·7, 54·4	<0·0001
Intakes of dish categories(/d)										
Grain dishes (serving)	4·66	4·42, 4·90	4·93	4·70, 5·17	0·10	4·68	4·59, 4·77	4·90	4·81, 5·00	0·0006
Vegetable dishes (serving)	4·48	4·03, 4·92	5·79	5·35, 6·23	<0·0001	5·12	4·94, 5·29	6·05	5·88, 6·22	<0·0001
Fish and Meat dishes (serving)	6·97	6·51, 7·44	6·14	5·69, 6·59	0·01	7·98	7·80, 8·17	6·55	6·36, 6·73	<0·0001
Milk (serving)	0·55	0·34, 0·76	1·49	1·28, 1·70	<0·0001	0·77	0·69, 0·85	1·42	1·34, 1·50	<0·0001
Fruits (serving)	0·57	0·39, 0·75	1·48	1·31, 1·65	<0·0001	0·51	0·45, 0·58	1·28	1·22, 1·34	<0·0001
Total energy intake (kcal)	1994	1917, 2070	2030	1955, 2105	0·50	2187	2156, 2218	2084	2054, 2115	<0·0001
Energy intake from snacks and alcoholic beverages (kcal)	263	232, 293	130	100, 160	<0·0001	315	302, 328	137	124, 150	<0·0001
Intakes of nutrients (/d)‡										
Protein (% energy)	14·4	13·9, 14·8	14·3	13·9, 14·8	0·95	14·7	14·5, 14·9	14·3	14·1, 14·5	0·001
Total fat (% energy)	23·8	22·8, 24·8	23·3	22·3, 24·3	0·50	25·4	25·0, 25·8	25·0	24·5, 25·4	0·14
Saturated fat (% energy)	6·0	5·7, 6·4	6·1	5·8, 6·4	0·81	6·5	6·3, 6·6	6·6	6·5, 6·7	0·23
Total carbohydrate (% energy)	54·9	53·6, 56·1	59·2	57·9, 60·4	<0·0001	51·6	51·1, 52·1	57·3	56·8, 57·8	<0·0001
Dietary fibre (g/1000 kcal)	6·4	6·0, 6·8	8·2	7·8, 8·6	<0·0001	6·5	6·3, 6·6	8·0	7·9, 8·2	<0·0001
Salt (g/1000 kcal)	5·25	4·99, 5·51	5·23	4·97, 5·48	0·91	5·28	5·18, 5·38	5·27	5·17, 5·37	0·90
Potassium (mg/1000 kcal)	1027	981, 1073	1244	1198, 1289	<0·0001	1054	1036, 1072	1218	1201, 1236	<0·0001
Women										
No. subjects	305		311			1305		1294		
Japanese Food Guide Spinning Top score	43·8	43·2, 44·4	57·6	57·0, 58·2	<0·0001	43·6	43·3, 43·9	57·5	57·2, 57·8	<0·0001
Intakes of dish categories(/d)										
Grain dishes (serving)	3·31	3·17, 3·45	3·62	3·48, 3·75	0·002	3·25	3·19, 3·32	3·64	3·58, 3·71	<0·0001
Vegetable dishes (serving)	4·71	4·38, 5·04	5·91	5·59, 6·24	<0·0001	4·80	4·65, 4·95	5·63	5·48, 5·78	<0·0001
Fish and Meat dishes (serving)	5·29	4·98, 5·59	5·32	5·02, 5·62	0·89	6·04	5·90, 6·18	5·20	5·06, 5·33	<0·0001
Milk (serving)	0·95	0·76, 1·13	1·64	1·45, 1·82	<0·0001	0·91	0·84, 0·99	1·69	1·61, 1·76	<0·0001
Fruits (serving)	0·81	0·67, 0·95	1·84	1·70, 1·97	<0·0001	0·68	0·62, 0·74	1·50	1·44, 1·56	<0·0001
Total energy intake (kcal)	1541	1492, 1590	1719	1670, 1767	<0·0001	1624	1602, 1647	1721	1699, 1744	<0·0001
Energy intake from snacks and alcoholic beverages (kcal)	143	126, 161	93	76, 110	<0·0001	182	173, 191	96	87, 105	<0·0001
Intakes of nutrients (/d)‡										
Protein (% energy)	15·0	14·6, 15·3	14·8	14·4, 15·2	0·55	15·5	15·4, 15·7	14·6	14·5, 14·8	<0·0001
Total fat (% energy)	25·4	24·5, 26·2	25·1	24·3, 26·0	0·69	27·7	27·3, 28·1	26·7	26·3, 27·1	0·001
Saturated fat (% energy)	6·5	6·2, 6·8	6·6	6·4, 6·9	0·57	7·2	7·1, 7·4	7·3	7·1, 7·4	0·73
Total carbohydrate (% energy)	57·0	56·0, 58·0	59·0	58·0, 60·0	0·005	53·8	53·4, 54·3	57·4	57·0, 57·9	<·0001
Dietary fibre (g/1000 kcal)	8·6	8·2, 9·0	9·8	9·5, 10·2	<0·0001	8·1	8·0, 8·3	9·3	9·2, 9·5	<0·0001
Salt (g/1000 kcal)	5·90	5·67, 6·13	5·50	5·27, 5·73	0·02	5·67	5·57, 5·77	5·58	5·48, 5·68	0·24
Potassium (mg/1000 kcal)	1272	1228, 1316	1445	1401, 1488	<0·0001	1246	1226, 1266	1387	1367, 1407	<0·0001

* Adjusted for residential block, population size of residential area, household size, one or more children aged under 15 years, occupation, BMI, smoking status, and physical activity·

† Individuals with median or higher scores were defined as a high quality of diet group and the remaining as a low quality of diet group·

‡ Nutrients whose tentative dietary goals for preventive lifestyle related diseases (DG) in the dietary reference intake for Japanese (2015) are defined.

Irrespective of sex and household income level, the proportion of individuals whose consumption was not meeting the recommended amounts for each dish category was higher in those with a low quality diet than those with a high quality diet (Table 4). As for fish and meat dishes, the proportion of individuals who consumed excess amounts of fish and meat dishes with a low quality diet was higher than those with a high quality diet. In men and women in the low-income subgroups, the proportions of individuals who consumed insufficient amounts of fish and meat dishes was also higher among those with a low quality diet compared with those with a high quality diet. The proportions of men in the low-income subgroup who consumed insufficient amounts of fish and meat dishes were 16 % and 4 % in those with low and high quality diets, respectively. The corresponding proportions in women in the low-income subgroup were 20 % and 8 %, respectively. However, the proportion of individuals who consumed insufficient amounts of fish and meat dishes among the higher-income subgroups did not differ according to diet quality. The multivariate adjusted ORs of not meeting the recommended amounts of all components of the Japanese Food Guide Spinning Top, except for energy intake from snacks and alcoholic beverages, were higher in the low-income subgroups than the higher-income ones. Additionally, the multivariate adjusted ORs of not meeting DG were higher in the low-income subgroups than the higher-income ones (Supplementary Table 2).

**Table 4.** Multivariate adjusted OR of not meeting the recommendation of the Japanese Food Guide Spinning Top according to quality of diet

	Low household income (<2 million yen/year)		Middle and high household income (≥2 million yen/year)	
	Low diet quality*	High diet quality*	Low diet quality*	High diet quality*
Men				
No. subjects	207	215	1174	1189
Grain dishes				
Insufficient (%)†	46·9	31·2	47·8	38·9
Adequate (%)‡	36·7	55·8	40·5	53·3
Excess (%)§	16·4	13·0	11·8	7·7
Multivariate adjusted OR of inadequate‖	2·28	1·00	1·78	1·00
95 % CI	1·52, 3·43	reference	1·51, 2·11	reference
Vegetable dishes				
Insufficient (%)†	62·3	39·5	52·6	33·4
Adequate (%)‡	37·7	60·5	47·4	66·6
Multivariate adjusted OR (95 % CI) of inadequate‖	2·66	1·00	2·22	1·00
95 % CI	1·75, 4·04	reference	1·86, 2·64	reference
Fish and Meat dishes				
Insufficient (%)†	15·9	4·2	5·9	4·4
Adequate (%)‡	20·3	37·7	18·7	32·9
Excess (%)§	63·8	58·1	75·4	62·7
Multivariate adjusted OR (95 % CI) of inadequate‖	2·50	1·00	2·25	1·00
95 % CI	1·58, 3·94	reference	1·85, 2·75	reference
Milk				
Insufficient (%)†	88·4	54·9	83.0	56·6
Adequate (%)‡	3·9	24·7	7·0	26·0
Excess (%)§	7·7	20·5	10·1	17·4
Multivariate adjusted OR (95 % CI) of inadequate‖	9·32	1·00	4·51	1·00
95 % CI	4·13, 21·00	reference	3·46, 5·89	reference
Fruits				
Insufficient (%)†	87·0	56·3	88·6	62·0
Adequate (%)‡	13·0	43·7	11·4	38·0
Multivariate adjusted OR (95 % CI) of inadequate‖	6·39	1·00	5·66	1·00
95 % CI	3·71, 11·01	reference	4·44, 7·20	reference
Total energy intake				
Insufficient (%)†	43·0	30·7	36·8	39·4
Adequate (%)‡	22·2	39·1	24·6	36·3
Excess (%)§	34·8	30·2	38·6	24·2
Multivariate adjusted OR (95 % CI) of inadequate‖	2·31	1·00	1·74	1·00
95 % CI	1·46, 3·66	reference	1·45, 2·09	reference
Energy intake from snacks and alcoholic beverages				
Adequate (%)‡	48·3	77·7	38·9	73·2
Excess (%)§	51·7	22·3	61·1	26·8
Multivariate adjusted OR (95 % CI) of inadequate‖	3·85	1·00	4·63	1·00
95 % CI	2·47, 5·99	reference	3·85, 5·57	reference
Women				
No. subjects	305	311	1305	1294
Grain dishes				
Insufficient (%)†	63·0	54·7	64·3	53·9
Adequate (%)‡	31·1	41·2	31·4	41·8
Excess (%)§	5·9	4·2	4·3	4·3
Multivariate adjusted OR (95 % CI) of inadequate‖	1·63	1·00	1·67	1·00
95 % CI	1·15, 2·30	reference	1·42, 1·98	reference
Vegetable dishes				
Insufficient (%)†	56·4	31·8	55·2	34·9
Adequate (%)‡	43·6	68·2	44·8	65·1
Multivariate adjusted OR (95 % CI) of inadequate‖	2·56	1·00	2·12	1·00
95 % CI	1·81, 3·63	reference	1·80, 2·50	reference
Fish and Meat dishes				
Insufficient (%)†	20·3	7·7	11·4	6·7
Adequate (%)‡	27·2	32·2	24·3	36·6
Excess (%)§	52·5	60·1	64·3	56·6
Multivariate adjusted OR (95 % CI) of inadequate‖	1·36	1·00	1·85	1·00
95 % CI	0·94, 1·95	reference	1·56, 2·21	reference
Milk				
Insufficient (%)†	80·0	48·6	79·5	47·0
Adequate (%)‡	6·6	26·7	7·2	29·9
Excess (%)§	13·4	24·8	13·3	23·1
Multivariate adjusted OR (95 % CI) of inadequate‖	5·87	1·00	5·53	1·00
95 % CI	3·40, 10·11	reference	4·32, 7·08	reference
Fruits				
Insufficient (%)†	82·6	44·4	84·4	55·6
Adequate (%)‡	17·4	55·6	15·6	44·4
Multivariate adjusted OR (95 % CI) of inadequate‖	5·63	1·00	4·62	1·00
95 % CI	3·80, 8·34	reference	3·77, 5·65	reference
Total energy intake				
Insufficient (%)†	51·8	27·3	43·0	31·5
Adequate (%)‡	33·1	53·7	38·5	52·5
Excess (%)§	15·1	19·0	18·5	16·1
Multivariate adjusted OR (95 % CI) of inadequate‖	2·35	1·00	1·83	1·00
95 % CI	1·67, 3·30	reference	1·56, 2·15	reference
Energy intake from snacks and alcoholic beverages				
Adequate (%)‡	72·1	86·2	64·0	83·8
Excess (%)§	27·9	13·8	36·0	16·2
Multivariate adjusted OR (95 % CI) of inadequate‖	2·61	1·00	3·12	1·00
95 % CI	1·70, 4·02	reference	2·57, 3·78	reference

* Individuals with median or higher scores were defined as a high quality of diet group and the remaining as a low quality of diet group.

† Insufficient was defined when the amount of intake was less than the recommended amount of the Japanese Food Guide Spinning Top.

‡ Adequate was defined when the amount of intake was equal to the recommended amount of the Japanese Food Guide Spinning Top.

§ Excess was defined when the amount of intake was more than the recommended amount of the Japanese Food Guide Spinning Top.

‖ Adjusted for residential block, population size of residential area, household size, one or more children aged under 15 years, occupation, BMI, smoking status, and physical activity.

## Discussion

In this population-based cross-sectional study using NHNS data in Japan, we found that high quality diet was closely related to female sex, older age, and higher household income. Our findings are consistent with previous findings from Western countries. In the US National Health and Nutrition Examination Survey, higher scores on the HEI-2005 (measure of diet quality in terms of adherence to the 2005 Dietary Guideline for the Americans) were related to older age, female sex, and higher household income among adults^(^^4^^)^. A cross-sectional study in the 1995 Australian National Nutrition Survey among individuals aged ≥18 years also showed that food variety differed by age, sex, and income^(^^5^^)^. In this previous study, older women in the higher-income households had higher scores regarding food variety^(^^5^^)^. In Western countries, higher diet quality scores were associated with lower risks of all-cause mortality, cardiovascular disease, cancer, type 2 diabetes, and neurodegenerative disease^(^^7^^)^. Furthermore, closer adherence to Japanese dietary guidelines was also associated with lower risks of total mortality and mortality from cardiovascular diseases^(^^8^^,^^9^^)^. The evidence suggests that improving diet quality might contribute to enhancing the life expectancy in adults.

With fish and meat dishes, lower-income individuals with a low quality diet were more likely to consume insufficient amounts of fish and meat dishes (16 % in men and 20 % in women) than those with a high quality diet (4 % in men and 8 % in women), although there were no large differences among higher-income individuals. Similarly, Korean adults with mild or severe food-insufficiency status consumed less meat, fish, eggs, and beans compared with those who were food-sufficient^(^^22^^)^. The contribution of fish and meat dishes (including meat, fish and shellfish), and eggs, to monetary diet cost (1022 Japanese yen per day) was highest (33 %) among Japanese Food Guide Spinning Top components in Japanese adults^(^^23^^)^. Given that the percentage of food products in the expenditure of the lower-income individuals is higher than the higher-income individuals on the basis of the Engel’s law^(^^24^^)^, we speculate that lower-income individuals are likely to reduce their fish and meat dishes in order to save money. However, given that the proportion of individuals who consumed excess amounts of fish and meat dishes was higher in the lower-income subgroup with a low quality diet than those with a high quality diet, especially among men, then there is need for dietary education to ensure that adequate amounts of fish and meat dishes are selected for intakes.

In the current study, individuals with a low quality diet consumed less vegetable dishes, milk, and fruits than those with a high quality diet, regardless of household income level. Of the Japanese Food Guide Spinning Top components, the ORs of inadequate consumption of milk and fruits were higher compared with other components. In some studies examining the associations between diet quality scores and intakes of food groups, in China^(^^25^^)^ and the United States^(^^26^^,^^27^^)^, individuals with lower diet quality scores consumed less vegetables and fruits compared with those with higher scores. This unfavourable aspect of the low quality diet in our study was in line with previous studies. Further research is needed to determine how low-income individuals could practice higher-quality diets without additional costs.

Previous cross-sectional studies in Western countries reported that low-income individuals consume high quantities of grains^(^^1^^)^. However, we found that low quality diet was related to less amount of grains regardless of household income level. Additionally, individuals with a low quality diet consumed a high amount of snacks and alcoholic beverages across all income levels. The different consumption of grains findings between Western countries and Japan might be partly due to country-specific dietary guideline definition of grains and snacks. For example, MyPlate, which was based on the 2015–2020 Dietary Guidelines for Americans, shows that grains consist of whole grains and refined grains, including not only white bread, white rice and maize flakes but also biscuits, cookies, cakes and pancakes^(^^28^^)^. In the Japanese Food Guide Spinning Top^(^^20^^)^, as well as NHNS, such foods as biscuits, cookies, cakes and pancakes are defined as snacks but not grains. If the Western studies applied the Japanese definition of grains, their findings might change. These differences in the definition of food groups suggest that caution is needed to interpret the findings concerning grain intake between countries.

What should the policy makers do to promote healthier diet in lower-income individuals? According to the consumer price index in Japan^(^^29^^)^, items with ≥20 % differences from 2000 to 2015 were vegetables, fruits, fish and shellfish, and milk and dairy products. The 2014 NHNS reported that individuals with higher frequency of giving up or not buying foods for financial reasons tended to focus on price when choosing foods^(^^14^^)^. The 2014 NHNS also reported that individuals with a higher frequency of giving up or not buying foods for financial reasons consumed less vegetables, fruits, fish and shellfish, and milk and dairy products as well as meats and eggs^(^^14^^)^. This evidence suggests that price adjustment for nutrient-rich foods (i.e. vegetables, fruits, fish and shellfish, meats, etc.) is needed to secure a stable supply of such foods. In Japan, the price of rice is adjusted according to the Act on Stabilization of Supply, Demand, and Prices of Staple Food (Act No. 113 of 1994). Thus, policymakers need to make efforts to ensure the supply of nutrient-rich foods to lower-income individuals.

The major strengths of this study include the use of a nationally representative data and adjustment for potentially confounding variables. Limitations of our study also warrant mentioning. First, we could not exclude the possibility of selection bias. Although the study household samples were randomly selected from nationally representative households in Japan, we excluded individuals with missing data on household income in the present analyses. However, the characteristics of individuals who were excluded in the current analysis were similar to those in the current analysis in terms of age, sex, BMI, size of household, and residential area. Second, dietary intake was assessed by 1-d semi-weighted household dietary record and might not represent long-term habitual intake. A 1-d dietary record may not accurately reflect an individual’s habitual intake. However, the nationally representative sample size of the NHNS population (*n* approximately 8000) from all over Japan, enables us to minimize the effect of intra-individual variation. Our findings were similar to the results of the 2010 NHNS, which showed that lower-income individuals had significantly lower amounts of vegetables than higher-income individuals (256 g *v*. 293 g in men and 270 g *v*. 305 g in women). Furthermore, similar 1-d dietary national surveys have also observed similar relationships between diet and socioeconomic status^(^^2^^)^. Third, we used the Japanese Food Guide Spinning Top score to assess diet quality. Although several studies^(^^8^^,^^9^^,^^30^^)^ in Japan used the score, the validity against dietary reference intakes has not been examined. In fact, more than 50 % of the current subjects were defined as those consuming excess amounts of fish and meat dishes. Recently, Hayabuchi *et al*. revised the number of servings for the Japanese Food Guide Spinning Top according to dietary reference intakes for Japanese (2015)^(^^31^^)^. They estimated that two servings more should be added to fish and meat dishes than the original spinning top^(^^31^^)^. When we applied the revised number of servings of fish and meat dishes for the standard, the proportion of individuals consuming excess amounts decreased to <50 %. Therefore, we suggest that the standard servings of the Japanese Food Guide Spinning Top should be revised. Fourth, the Japanese Food Guide Spinning Top score takes fish and meat as one food group (fish and meat dishes). Fish is rich in *n*-3 polyunsaturated fatty acids, whereas red meat, including beef and pork, contains saturated fatty acids^(^^19^^)^. A randomized controlled trial indicated that the quality of fat might have a primary impact on cardiovascular disease risk^(^^32^^)^. However, Kurotani *et al*. found similar associations of mortality with the Japanese Food Guide Spinning Top scores to those with the modified scores considering the quality of fat^(^^9^^)^. Fifth, the Japanese Food Guide Spinning Top score does not separate grain dishes according to the composition of fibre. However, the dietary guidelines in other countries have similar situations. For example, MyPlate also shows that grains consist of both whole grains and refined grains^(^^28^^)^. Finally, we cannot completely rule out the effects of confounding by residual and unmeasured variables.

## Conclusion

In conclusion, this cross-sectional study of Japanese representative samples showed that the diet quality of Japanese adults differed by age, sex, and household income level. Low-income individuals with a low quality diet were less likely to consume the recommended amounts of the Japanese Food Guide Spinning Top, especially milk and fruits, compared with higher-income individuals. This suggests that a different approach for diet quality improvement might be needed according to household income level. To reduce health disparities, policy makers should ensure services to improve the food environment including securing a stable supply of nutrient-rich foods by reducing the price of those foods.

## Supplementary material

For supplementary material accompanying this paper visit https://doi.org/10.1017/S1368980019002088. click here to view supplementary material
