# Supplementary material for: Diet quality of Japanese adults with respect to age, sex, and income level in the National Health and Nutrition Survey, Japan
Source: Public Health Nutr. 2019 Nov 18;23(5):821–32. doi: 10.1017/S1368980019002088 (PMC7282861; doi:10.1017/S1368980019002088)
Supplement: Supplementary file 1 [file S1368980019002088sup001.doc]

# Supplementary Table 1

# Calculation of the Japanese Food Guide Spinning Top score

| The Japanese Food Guide Spinning Top component | Number of servings for 0 points | | Standard for continuous scoring of 0 to 10 points *^a^* | Number of servings for 10 points |
| --- | --- | --- | --- | --- |
| Grain dishes (serving/d) *^b^* | 0 or ≥11.0 *^c^* | | 0< to <3.5 or 5.5< to <11.0 *^c^* | 3.5–5.5 *^c^* |
|  | 0 or ≥15.0 *^d^* | | 0< to <4.5 or 7.5< to <15.0 *^d^* | 4.5–7.5 *^d^* |
|  | 0 or ≥17.0 *^e^* | | 0< to <5.5 or 8.5< to <17.0 *^e^* | 5.5–8.5 *^e^* |
|  |  | |  |  |
| Vegetable dishes (serving/d) *^f^* | 0 | | 0< to <4.5 *^c d^* | ≥4.5 *^c d^* |
|  |  | | 0< to <5.5 *^e^* | ≥5.5 *^e^* |
|  |  | |  |  |
| Fish and meat dishes (serving/d) *^g^* | 0 or ≥9.0 *^c^* | | 0< to <2.5 or 4.5< to <9.0 *^c^* | 2.5–4.5 *^c^* |
|  | 0 or ≥11.0 *^d^* | | 0< to <2.5 or 5.5< to <11.0 *^d^* | 2.5–5.5 *^d^* |
|  | 0 or ≥13.0 *^e^* | | 0< to <3.5 or 6.5< to <13.0 *^e^* | 3.5–6.5 *^e^* |
|  |  | |  |  |
| Milk (serving/d) *^h^* | 0 or ≥5.0 *^c d^* | | 0< to <1.5 or 2.5< to <5.0 *^c d^* | 1.5–2.5 *^c d^* |
|  | 0 or ≥7.0 *^e^* | | 0< to <1.5 or 3.5< to <7.0 *^e^* | 1.5–3.5 *^e^* |
|  |  | |  |  |
| Fruits (serving/d) *^i^* | 0 | | 0< to <1.5 | ≥1.5 |
|  |  | |  |  |
| Total energy (kcal/d) | ≥4000 *^c^* | | <1400 or 2000< to <4000 *^c^* | 1400–2000 *^c^* |
|  | ≥4800 *^d^* | | <2000 or 2400< to <4800 *^d^* | 2000–2400 *^d^* |
|  | ≥6000 *^e^* | | <2400 or 3000< to <6000 *^e^* | 2400–3000 *^e^* |
|  |  | |  |  |
| Snacks and alcoholic beverages (kcal/d) | | ≥400 | 200< to <400 | 0–200 |
| The Japanese Food Guide Spinning Top score (0–70) | Grain dishes score + vegetable dishes score + fish and meat dishes score + milk score + fruits score + total energy score + snacks and alcoholic beverages score | | | |

*^a^* If an individual consumed less than the recommended amount of servings or energy, the score was calculated using the following formula: 10 × (the consumed amount of servings or energy)/(the lower limit of the recommended amount). If an individual consumed more than the recommended amount of servings or energy, the score was calculated using the following formula: 10 − 10 × [(the consumed amount of servings or energy) − (the upper limit of the recommended amount)]/(the upper limit of the recommended amount).

*^b^* Grain dishes included rice, bread and noodles. One serving of a grain dish is composed of about 40 g carbohydrates.

*^c^* 70 y or older in men and 70 y or older or 18–69 y with sedentary in women

*^d^* 18–69 y with sedentary in men and 18–69 y with moderate physical activity in women

*^e^* 18–69 y with moderate physical activity in men

*^f^* Vegetable dishes included vegetables, mushrooms, potatoes and seaweed. In one serving of a vegetable dish, the main ingredient weighs about 70 g.

*^g^* Fish and meat dishes included meat, fish, eggs and soybeans. One serving of fish and meat dish contains about 6 g protein.

*^h^* Milk was milk and milk products. One serving of milk contains about 100 mg calcium.

*^i^* In one serving of fruits, the main ingredient weighs about 100 g.

**Supplementary Table 2** Multivariate adjusted odds ratios (OR) of not meeting DG according to quality of diet by sex

|  | Low household income  (<2 million yen/year) | |  | Middle and high household income  (≥2 million yen/year) | |
| --- | --- | --- | --- | --- | --- |
|  | Low diet quality *^a^* | High diet quality *^a^* |  | Low diet quality *^a^* | High diet quality *^a^* |
| *Men* |  |  |  |  |  |
| Number of subjects | 207 | 215 |  | 1174 | 1189 |
| Protein (% energy) |  |  |  |  |  |
| Less than DG *^b^* (%) | 37.7 | 32.1 |  | 32.4 | 30.5 |
| DG (%) | 56.0 | 65.1 |  | 62.3 | 66.9 |
| More than DG (%) | 6.3 | 2.8 |  | 5.4 | 2.6 |
| Multivariate adjusted OR (95% CI) of not meeting DG *^c^* | 1.45 (0.96-2.19) | 1.00 (reference) |  | 1.12 (0.94-1.34) | 1.00 (reference) |
| Total fat (% energy) |  |  |  |  |  |
| Less than DG (%) | 32.4 | 31.2 |  | 24.5 | 25.7 |
| DG (%) | 46.9 | 56.7 |  | 48.8 | 49.5 |
| More than DG (%) | 20.8 | 12.1 |  | 26.8 | 24.9 |
| Multivariate adjusted OR (95% CI) of not meeting DG *^c^* | 1.43 (0.96-2.13) | 1.00 (reference) |  | 1.03 (0.87-1.21) | 1.00 (reference) |
| Saturated fat (% energy) |  |  |  |  |  |
| DG (%) | 66.2 | 69.3 |  | 63.5 | 59.0 |
| More than DG (%) | 33.8 | 30.7 |  | 36.5 | 41.0 |
| Multivariate adjusted OR (95% CI) of not meeting DG *^c^* | 1.11 (0.72-1.71) | 1.00 (reference) |  | 0.86 (0.72-1.02) | 1.00 (reference) |
| Total carbohydrate (% energy) |  |  |  |  |  |
| Less than DG (%) | 29.5 | 11.2 |  | 44.8 | 19.6 |
| DG (%) | 56.0 | 66.1 |  | 46.3 | 61.6 |
| More than DG (%) | 14.5 | 22.8 |  | 8.9 | 18.8 |
| Multivariate adjusted OR (95% CI) of not meeting DG *^c^* | 1.48 (0.98-2.24) | 1.00 (reference) |  | 1.86 (1.57-2.21) | 1.00 (reference) |
| Dietary fiber |  |  |  |  |  |
| Less than DG (%) | 85.0 | 72.1 |  | 82.9 | 75.0 |
| DG (%) | 15.0 | 27.9 |  | 17.1 | 25.0 |
| Multivariate adjusted OR (95% CI) of not meeting DG *^c^* | 2.20 (1.31-3.70) | 1.00 (reference) |  | 1.52 (1.22-1.88) | 1.00 (reference) |
| Salt *^d^* |  |  |  |  |  |
| DG (%) | 34.8 | 27.9 |  | 24.2 | 22.0 |
| More than DG (%) | 65.2 | 72.1 |  | 75.8 | 78.1 |
| Multivariate adjusted OR (95% CI) of not meeting DG *^c^* | 0.76 (0.49-1.17) | 1.00 (reference) |  | 0.92 (0.76-1.13) | 1.00 (reference) |
| Potassium |  |  |  |  |  |
| Less than DG (%) | 869.5 | 72.1 |  | 79.8 | 74.6 |
| DG (%) | 13.5 | 27.9 |  | 20.2 | 25.4 |
| Multivariate adjusted OR (95% CI) of not meeting DG *^c^* | 2.48 (1.44-4.26) | 1.00 (reference) |  | 1.24 (1.01-1.53) | 1.00 (reference) |
| *Women* |  |  |  |  |  |
| Number of subjects | 305 | 311 |  | 1305 | 1294 |
| Protein (% energy) |  |  |  |  |  |
| Less than DG^2^ (%) | 33.8 | 26.4 |  | 22.3 | 25.0 |
| DG (%) | 57.4 | 70.7 |  | 69.0 | 73.2 |
| More than DG (%) | 8.9 | 2.9 |  | 8.7 | 1.9 |
| Multivariate adjusted OR (95% CI) of not meeting DG *^c^* | 1.71 (1.22-2.42) | 1.00 (reference) |  | 1.15 (0.96-1.37) | 1.00 (reference) |
| Total fat (% energy) |  |  |  |  |  |
| Less than DG (%) | 26.9 | 23.2 |  | 17.0 | 17.4 |
| DG (%) | 41.6 | 53.1 |  | 45.1 | 51.8 |
| More than DG (%) | 31.5 | 23.8 |  | 37.9 | 30.8 |
| Multivariate adjusted OR (95% CI) of not meeting DG *^c^* | 1.69 (1.21-2.36) | 1.00 (reference) |  | 1.33 (1.14-1.56) | 1.00 (reference) |
| Saturated fat (% energy) |  |  |  |  |  |
| DG (%) | 60.7 | 59.2 |  | 49.6 | 48.8 |
| More than DG (%) | 39.3 | 40.8 |  | 50.4 | 51.2 |
| Multivariate adjusted OR (95% CI) of not meeting DG *^c^* | 0.90 (0.63-1.27) | 1.00 (reference) |  | 0.96 (0.81-1.12) | 1.00 (reference) |
| Total carbohydrate (% energy) |  |  |  |  |  |
| Less than DG (%) | 24.6 | 10.6 |  | 34.0 | 16.6 |
| DG (%) | 55.7 | 68.2 |  | 54.8 | 66.7 |
| More than DG (%) | 19.7 | 21.2 |  | 11.2 | 16.7 |
| Multivariate adjusted OR (95% CI) of not meeting DG *^c^* | 1.76 (1.27-2.54) | 1.00 (reference) |  | 1.66 (1.41-1.95) | 1.00 (reference) |
| Dietary fiber |  |  |  |  |  |
| Less than DG (%) | 80.7 | 59.2 |  | 82.5 | 65.1 |
| DG (%) | 19.3 | 40.8 |  | 17.5 | 34.9 |
| Multivariate adjusted OR (95% CI) of not meeting DG *^c^* | 2.60 (1.77-3.82) | 1.00 (reference) |  | 2.41 (1.99-2.92) | 1.00 (reference) |
| Salt *^d^* |  |  |  |  |  |
| DG (%) | 34.8 | 22.8 |  | 30.0 | 21.1 |
| More than DG (%) | 65.3 | 77.2 |  | 70.0 | 78.9 |
| Multivariate adjusted OR (95% CI) of not meeting DG *^c^* | 0.59 (0.40-0.86) | 1.00 (reference) |  | 0.67 (0.55-0.80) | 1.00 (reference) |
| Potassium |  |  |  |  |  |
| Less than DG (%) | 80.7 | 60.5 |  | 79.9 | 64.0 |
| DG (%) | 19.3 | 39.6 |  | 20.1 | 36.0 |
| Multivariate adjusted OR (95% CI) of not meeting DG *^c^* | 2.44 (1.65-3.59) | 1.00 (reference) |  | 2.11 (1.75-2.54) | 1.00 (reference) |

*^a^* Individuals with median or higher scores were defined as a high quality of diet group and the remaining as a low quality of diet group.

*^b^* DG was a tentative dietary goal for preventing lifestyle related diseases in the Dietary Reference Intakes for Japanese (2015).

*^c^* Adjusted for residential block, population size of residential area, household size, one or more children aged under 15 years, occupation, body mass index, smoking status, and physical activity.

*^d^* Salt = sodium (mg)×2.54/1000
